# Current Treatment for Venom-Induced Consumption Coagulopathy Resulting from Snakebite

**DOI:** 10.1371/journal.pntd.0003220

**Published:** 2014-10-23

**Authors:** Kalana Maduwage, Geoffrey K. Isbister

**Affiliations:** 1 School of Medicine and Public Health, University of Newcastle, New South Wales, Australia; 2 South Asian Clinical Toxicology Research Collaboration, Peradeniya, Sri Lanka; 3 Department of Biochemistry, Faculty of Medicine, University of Peradeniya, Peradeniya, Sri Lanka; 4 Department of Clinical Toxicology and Pharmacology, Calvary Mater Newcastle, Newcastle, New South Wales, Australia; Faculty of Medicine, University of Kelaniya, Sri Lanka

## Abstract

Venomous snakebite is considered the single most important cause of human injury from venomous animals worldwide. Coagulopathy is one of the commonest important systemic clinical syndromes and can be complicated by serious and life-threatening haemorrhage. Venom-induced consumption coagulopathy (VICC) is the commonest coagulopathy resulting from snakebite and occurs in envenoming by Viperid snakes, certain elapids, including Australian elapids, and a few Colubrid (rear fang) snakes. Procoagulant toxins activate the clotting pathway, causing a broad range of factor deficiencies depending on the particular procoagulant toxin in the snake venom. Diagnosis and monitoring of coagulopathy is problematic, particularly in resource-poor countries where further research is required to develop more reliable, cheap clotting tests. MEDLINE and EMBASE up to September 2013 were searched to identify clinical studies of snake envenoming with VICC. The UniPort database was searched for coagulant snake toxins. Despite preclinical studies demonstrating antivenom binding toxins (efficacy), there was less evidence to support clinical effectiveness of antivenom for VICC. There were no placebo-controlled trials of antivenom for VICC. There were 25 randomised comparative trials of antivenom for VICC, which compared two different antivenoms (ten studies), three different antivenoms (four), two or three different doses or repeat doses of antivenom (five), heparin treatment and antivenom (five), and intravenous immunoglobulin treatment and antivenom (one). There were 13 studies that compared two groups in which there was no randomisation, including studies with historical controls. There have been numerous observational studies of antivenom in VICC but with no comparison group. Most of the controlled trials were small, did not use the same method for assessing coagulopathy, varied the dose of antivenom, and did not provide complete details of the study design (primary outcomes, randomisation, and allocation concealment). Non-randomised trials including comparison groups without antivenom showed that antivenom was effective for some snakes (e.g., Echis), but not others (e.g., Australasian elapids). Antivenom is the major treatment for VICC, but there is currently little high-quality evidence to support effectiveness. Antivenom is not risk free, and adverse reactions can be quite common and potentially severe. Studies of heparin did not demonstrate it improved outcomes in VICC. Fresh frozen plasma appeared to speed the recovery of coagulopathy and should be considered in bleeding patients.

## Introduction

Venomous snakebite is considered to be the single most important cause of human injury from any kind of venomous or poisonous animal worldwide. Envenoming and deaths resulting from snakebite are a particularly important public health problem in the tropical world, with the highest burden in rural areas of South Asia, Southeast Asia, and sub-Saharan Africa [1]. Coagulopathy is the commonest important, systemic clinical syndrome caused by snake envenoming in the world, and venom-induced consumption coagulopathy (VICC) is the most clinically important coagulopathy, because it can be complicated by serious and life-threatening haemorrhage [2].

## Methods

We searched MEDLINE from 1946 and EMBASE from 1947 to September 2013 and included any clinical studies of snake envenoming with VICC which provided information on treatment, including antivenom. The following keywords were used: “snakebite”, “snake envenoming/envenomation”, “coagulopathy”, “bleeding”, “haemorrhage”, “antivenom”, “heparin”, and “treatment”. Reference lists of identified articles were searched to find additional publications. Only articles in English were reviewed. The UniPort database (www.uniport.org) was also used for information on isolated toxins from snake venoms with coagulant actions. We identified a total of 1,355 studies of which 95 were included for review. There were 25 randomised comparative trials, 13 non-randomised comparative trials, and a large number of observational clinical studies which discussed the effectiveness of treatments for VICC.

## Venom-Induced Consumption Coagulopathy (VICC)

Various terms have been used to refer to the consumption coagulopathy following snake envenoming, including disseminated intravascular coagulation (DIC), defibrination syndrome, and procoagulant coagulopathy [3]. More recently, the term “venom-induced consumption coagulopathy” has been introduced because it provides a more general description of the coagulopathy [4]. VICC can occur in envenoming by Viperid snakes, certain elapids, including Australian elapids [2], and a few Colubrid (rear fang) snakes [5]. A list of the major snake species that cause VICC is included in Table 1.

**Table 1:** Summary of snakes known to cause venom-induced consumption coagulopathy, the procoagulant toxin, and the factor deficiencies that have been reported (with permission from WikiToxin).

Snake species	Common name	Distribution	Procoagulant Toxins	VICC Testing	Factor Deficiencies	References
*Daboia russelii*	Russell's viper	Asia	FX, FV activators	WBCT20, CT, fibrinogen, clotting factor studies	Fibrinogen, FV, FX	Phillips [7], Isbister [16]
*Daboia russelii siamensis*	Eastern Russell's viper, Siamese Russell's viper	Asia	FX, FV activators	PT, non-clotting blood	Fibrinogen, FV, FX	Than [88], Tin Na [78]
*Hypnale hypnale*	Hump-nosed pit vipers	Asia	Unknown? TLE	WBCT20, PT, aPTT, clotting factor studies, D-Dimer	Fibrinogen, FVIII	Maduwage [13]
*Echis carinatus*	Saw scaled viper	Asia	PTA	WBCT20	NR	Kularatne [11]
*Calloselasma rhodostoma*	Malayan pit viper	Asia	TLE	VCT >30 minutes, fibrinogen, FDP, clotting factor studies	Fibrinogen	Kulapongs [89], Warrell [70]
*Trimeresurus albolabris*	White-lipped green pit viper	Asia	TLE	Fibrinogen, FDP, fibrinopeptide A, plasminogen	Fibrinogen	Hutton [90], Rojnuckarin [91]
*Trimeresurus macrops*	Large-eyed pitviper (green pitviper)	Asia	TLE	Fibrinogen, FDP, fibrinopeptide A, plasminogen	Fibrinogen	Rojnuckarin [91]
*Trimeresurus stejnegeri*	Bamboo pitviper, Chinese tree viper	Asia	TLE, plasminogen activator	Fibrinogen, FDP, AT-III	Fibrinogen	Li [92]
*Rhabdophis subminiatus*	Red-necked keelback	Asia	?	Fibrinogen, FDP	Fibrinogen	Li [92]
*Rhabdophis tigrinus*	Tiger keelback	Asia	?	PT, aPTT, Fibrinogen, FDP	Fibrinogen	Mori [93]
*Pseudonaja spp.*	Brown snake	Australia	PTA	PT, aPTT, clotting factor studies, D-dimer	Fibrinogen, FII, FV, FVIII	Isbister [3]
*Notechis scutatus*	Tiger snake	Australia	PTA	PT, aPTT, clotting factor studies, D-dimer	Fibrinogen, FII, FV, FVIII	Isbister [3]
*Tropidechis carinatus*	Rough-scaled snake	Australia	PTA	PT, aPTT, clotting factor studies, D-dimer	Fibrinogen, FII, FV, FVIII	Isbister [3]
*Hoplocephalus spp.*	Broad-headed snakes	Australia	PTA	PT, aPTT, clotting factor studies, D-dimer	Fibrinogen, FII, FV, FVIII	Isbister [3]
*Oxyuranus scutellatus*	Coastal taipan	Australasia	PTA	PT, aPTT, clotting factor studies, D-dimer	Fibrinogen, FII, FV, FVIII	Isbister [3], Lalloo [94]
*Bothrops atrox*	Common Lancehead	South America	TLE, FX, FV, activators	PT, aPTT, D-dimer, FDP	Fibrinogen	Pardal [60]
*Bothrops asper*	Lancehead, Terciopelo	South America	TLE, PTA	PT, aPTT, clotting factor studies, D-dimer	Fibrinogen, FII, FV	Barrantes [95], Otero-Patino [59]
*Bothrops jararaca*	Jararaca	South America	TLE, PTA, FX activator	Fibrinogen, clotting factor studies	Fibrinogen, FII, FV, FVIII	Kamiguti [96], Jorge [71]
*Lachesis* spp.	Bushmasters	Central America	TLE	Fibrinogen, D-dimer, α2-antiplasmin, FDP	Fibrinogen	Pardal [60]
*Crotalus durissus*	South American rattlesnake	Central and South America	TLE	PT, aPTT, clotting factor studies, D-dimer, FDP	Fibrinogen, FII, FV	Sano-Martin [97], Kamiguti [98]
*Crotalus atrox*	Western diamondback rattlesnake	North America	TLE	PT, aPTT, Fibrinogen	Fibrinogen	Budzynski [99]
*Crotalus adamanteus*	Eastern diamondback rattlesnake	North America	TLE	PT, aPTT, fibrinogen, D-dimer, FDP, antiplasmin III	Fibrinogen, D-dimer (normal)	Kitchens [100]
*Crotalus molossus molossus*	Black-tailed rattlesnake	North America	? TLE	PT, fibrinogen, FDP	Fibrinogen	Hardy [101]
*Crotalus horridus*	Timber rattlesnake	North America	TLE	Fibrinogen, FDP	Fibrinogen	Hasiba [102]
*Crotalus helleri*	Southern Pacific rattlesnake	North America	TLE	PT, fibrinogen	Fibrinogen	Bush [103]
*Vipera aspis*	European asp/Asp viper	Europe	FX activator	PT, aPTT, fibrinogen, D-dimer	Fibrinogen	Boels [104], Petite [105]
*Vipera berus*	Common European viper	Europe		PT, aPTT, fibrinogen, D-dimer	Fibrinogen	Boels [104]
*Vipera ammodytes ammodytes*	Horned viper	Europe		PT, aPTT, fibrinogen, D-dimer	Fibrinogen	Luksic [106]
*Atheris squamigera*	Green bush viper	Africa	TLE	aPTT, fibrinogen	Fibrinogen	Mebs [107]
*Atheris chlorechis*	Western bush viper	Africa	TLE	PT, aPTT, fibrinogen	Fibrinogen	Top [108]
*Atheris nitschei*	Great lakes bush viper	Africa	TLE	PT, aPTT, fibrinogen, D-dimer	Fibrinogen	Hatten [109]
*Cerastes cerastes*	Saharan horned viper	Africa/Middle East	TLE	PT, aPTT, fibrinogen, D-dimer, factor V	Fibrinogen, FV	Lifshitz [110], Schneemann [111]
*Cerastes vipera*	Sahara sand viper	Africa/Middle East	TLE (cerastobin)	PT, aPTT, fibrinogen, D-dimer	Fibrinogen	Lifshitz [112]
*Proatheris superciliaris*	Lowland viper	Africa		PT, aPTT, fibrinogen, D-dimer	Fibrinogen	Valenta [113]
*Bitis arietans*	African puff adders	Africa	TLE	Fibrinogen, PT, clotting factor studies	Fibrinogen	Jennings [114], Warrell [115], Lavonas [116]
*Bitis gabonica*	Gaboon viper	Africa	TLE (Gabonase)	Fibrinogen, PT, clotting factor studies	Fibrinogen	McNally [117]
*Echis coloratus*	Painted carpet viper	Africa	PTA	Fibrinogen, FDP, PT	Fibrinogen,? FII, FV, FVIII	Porath [118] Mann [73]
*Echis ocellatus*	West African carpet viper	Africa	PTA	WBCT20, fibrinogen, clotting factor studies	Fibrinogen, FII, FV, FVIII	Warrell [8]
*Echis pyramidum*	Northeast African carpet viper	Africa	PTA	Fibrinogen, PT, clotting factor studies	Fibrinogen, FII, FV, FVIII	Mion [22], Gillissen [119]
*Dispholidus typus*	Boomslang	Africa	SVMP*	PT, aPTT, fibrinogen, FDP, thromboelastography	Fibrinogen	Aitchison [5]

aPTT – activated partial thromboplastin time, CT – clotting time, VCT – venous clotting time, FDP – fibrinogen degradation products, PLA_2_ – phospholipase A_2_, PT – prothrombin time, TLE – thrombin like enzymes, WBCT – whole blood clotting time, WBCT20 – 20 minutes whole blood clotting time, FII – factor II, FV – factor V, FX – factor X, FDP – fibrinogen degradation products; PTA – prothrombin activator; SVMP – snake venom metalloproteinase; NR – not reported;

* A SVMP has been isolated from *D. typus* venom but its function (? PTA, FX activator, TLE) is unclear and only fibrinogen has been measured in patients.

**Figure 1:**
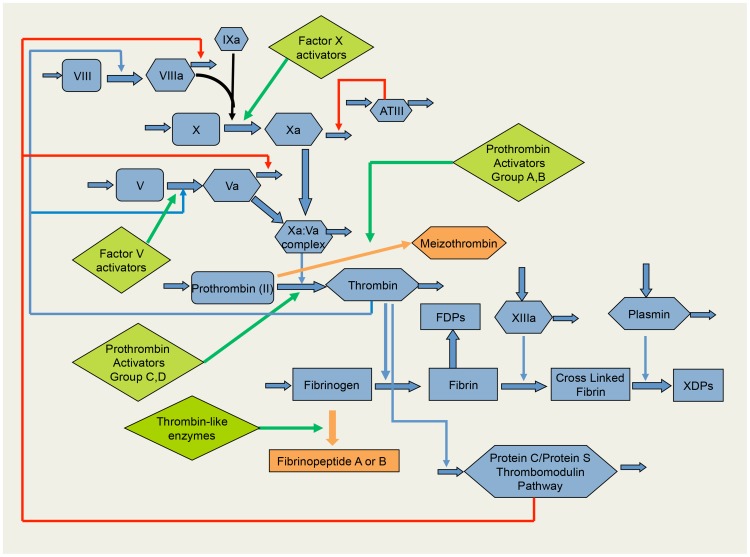
Diagram of the clotting pathway showing the major clotting factors (blue) and their role in the activation of the pathway and clot formation. The four major groups of snake toxins that activated the clotting pathway are in green and the intermediate or incomplete products they form are indicated in dark red. There are four major types of prothrombin activators, which either convert thrombin to form the catalytically active meizothrombin (Group A and B) or to thrombin (Group C and D).

VICC results from the activation of the clotting pathway by procoagulant toxins in the venom. The snake venom components that act on the coagulation system are classified according to the part of the coagulation pathway where they act and include factor V activators, factor X activators, prothrombin activators, and thrombin-like enzymes (TLEs) or fibrinogenases (Figure 1) [6]. The severity, duration, and type of consumption coagulopathy differ depending on the type of procoagulant toxin. Table 1 provides more detailed information on the clotting factor deficiencies in VICC for different snake groups. Almost all of these toxins cause activation of one or more clotting factors and lead to low or undetectable concentrations of fibrinogen following envenoming [2]. Thrombin-like enzymes or fibrinogenases generally cleave either the α-chain or the β-chain of fibrinogen to give fibrinopeptide A or B, which results in the consumption of fibrinogen without forming fibrin [4]. Therefore these toxins do not strictly activate the entire clotting pathway, but result in low or undetectable fibrinogen concentration, often with normal levels of the other clotting factors. In contrast, toxins that act higher up the clotting pathway, such as factor X activators or prothrombin activators, result in multiple factor deficiencies such as those occurring with Australian elapids [2], Russell's viper [7], and *Echis* spp. [8].

The diagnosis and monitoring of VICC requires coagulation studies and clotting times [9]. The majority of these tests are rarely available where most cases of snake envenoming occur. Internationally, the most commonly used test is the 20-minute whole blood clotting test (WBCT20) [8], [10]–[15]. However, the reliability of the WBCT20 as a diagnostic test has come into question for the diagnosis of Russell's viper envenoming [16]. There is no standardisation of the WBCT, including the duration of the test, the type of glass tube used for the test, and the procedure. The duration of the WBCT ranges from 10 minutes [17] in some studies to 30 minutes in others [18]–[20]. In other parts of the world more routine clotting tests, such as the prothrombin time (PT; international normalised ratio, INR), activated partial thromboplastin time (aPTT), and thrombin clotting time (TCT), are used [2], [21]–[23].

Many patients with VICC may exhibit minimal clinical features other than bleeding from the bite site or cannula site. However, some patients develop bleeding from the gums, gastrointestinal tract bleeding (clinically manifesting as melaena or haematemesis), and haematuria [7]. This is usually caused by snakes with venoms that also contain haemorrhagins such as *Echis* spp. [22], [24] and *Bothrops* spp. [25]. More severe bleeding includes intracranial haemorrhage, which is often fatal, and bleeding associated with trauma. Bleeding into the pituitary gland has also been reported following viper envenoming and produces subtle or delayed clinical signs compatible with Sheehan's syndrome [26], [27].

This review will focus on the effectiveness of various treatments for VICC based on the evidence from clinical studies, but will not cover venom-induced thrombocytopenia, [28], [29], snakebite-associated thrombotic microangiopathy [2], or anticoagulant coagulopathy [30].

## Antivenom

### Antivenoms and their mechanism of action

Antivenom is the recommended standard treatment for snake envenoming. Antivenoms consist of polyclonal antibodies to the toxins in snake venoms [31]. They may be whole immunoglobulins (IgG) or fractionated IgG, either F(ab′)_2_ or Fab [32]. The antibodies are produced in animals, including horse, sheep, goats, and rabbits. The animals are injected with the snake venom so that they mount an immune response and produce antibodies to that venom [33], [34]. The polyclonal nature of antivenom means that they are able to neutralise multiple venom components [32]. Monovalent antivenoms are raised against one species of snakes, while polyvalent snake antivenoms are produced by immunizing with venoms from more than one species of snake [33].

The efficacy of antivenom is best defined as its ability to bind venom components or toxins [35], while the effectiveness of antivenom is its ability to prevent or reverse the effects of envenoming in humans. There are a number of proposed mechanisms by which the binding of antivenom to venom results in prevention or reversal of envenoming. Antivenom can potentially block the active site of a toxin or bind to a toxin to prevent it interacting with its substrate (steric hindrance) to neutralise the toxin. Antivenom-venom complex formation in the central compartment may prevent the distribution of toxins to the target tissues (e.g., nervous system) or cause the redistribution of toxins from their target tissues back to the vascular compartment [32], [36]–[38]. Finally, antivenom can increase the elimination of toxins from the circulation and body. In the case of VICC, the toxins act in the central compartment, so antivenom must either bind to the toxins in the blood, and therefore prevent the action of the toxins, or increase the elimination of toxins.

Numerous studies have demonstrated that antivenom can bind to procoagulant toxins and prevent their effects in vitro if the antivenom and venom are pre-mixed [39]–[43]. Despite antivenom being efficacious and binding to the multiple toxins in the venom, there are a number of reasons that it may not be effective [35]. The most important being that irreversible toxic effects cannot be reversed by antivenom binding to toxins after the damage has occurred, such as clotting factor deficiencies resulting from VICC [9]. For antivenom to be effective against such irreversible effects, it must be administered early, so it can bind with toxins before they distribute to their target sites and cause irreversible toxicity.

Procoagulant toxins act in the central compartment (circulation), making their onset of action relatively rapid. Once they have activated the clotting pathway and clotting factors have been consumed, this process is irreversible until further clotting factors can be re-synthesized. Antivenom can only be effective in preventing the onset of VICC if it binds the procoagulant toxins prior to the clotting pathway being activated. A semi-mechanistic systems model of the coagulation cascade has been used to simulate the effects of Australasian elapid venoms on the clotting pathway and shows that antivenom needs to be administered within 15 to 30 minutes to prevent or even partially prevent VICC occurring [44]–[46]. However, the duration of VICC can be days for some snakes such as *Echis* spp., so the administration of antivenom will potentially bind the active procoagulant toxins, allowing the clotting factors to recover. Antivenom will therefore be clinically effective in shortening the duration of VICC and reducing the risk of bleeding.

Antivenoms are not without risk because administration of foreign proteins in the antivenoms can cause systemic hypersensitivity reactions (SHR) [47]. Early SHR include skin-only SHR, and anaphylaxis and severe anaphylaxis have been reported. Delayed reactions can also occur, and are referred to as serum sickness.

### Clinical studies of antivenom

Our literature review did not identify any placebo randomised control trials of snake antivenom for VICC. There were 25 randomised comparative trials [24], [48]–[71] of antivenom for VICC, which compared two different antivenoms (ten studies), three different antivenoms (four studies), two or three different doses or repeat doses of antivenom (five studies), heparin treatment and antivenom (five studies), and intravenous immunoglobulin treatment and antivenom (one study) (Table 2). There were a further 13 studies [8], [9], [20]–[22], [72]–[79] which compared two groups in which there was no randomisation, including studies with historical controls (Table 3). There have been numerous observational studies of patients with VICC given antivenom, but with no comparison group.

**Table 2:** Summary of the randomised comparative trials of treatment for VICC, including antivenom and heparin with details of study size, design, and outcomes.

Study	Numberin each arm	Snake species	Trial Arms	Blinded	Randomisation method	Allocation concealed	AV dose defined	Primary outcome	VICC measures	Conclusion†
Abubakar, 2010	194/206	*Echis ocellatus*	2 AV	Yes	Yes	Good	Yes	Yes	WBCT20	No difference between antivenoms (neither inferior)
Ariaratnam, 2001	23/20	*Daboia russelii*	2 AV	No	Yes	Good	Yes	No	WBCT20	No difference but multiple outcomes
Meyer, 1997	22/17	*E. ocellatus*	2 AV	No	Yes	Nil	Yes	No	WBCT20	No difference in restoration of clotting function
Otero, 1999	25/28	*Bothrops Porthidium* sp.	2 AV	Yes	Nil	Nil	Yes Varied*	Partially, defines 2	WBCT15/30	No difference for either outcome
Otero, 1996	20/19	*B. atrox*	2 AV	Yes	Nil	Good	Yes Varied*	No	WBCT15/30	No difference but no clearly defined outcomes
Otero, 2006	34/33	*B. asper*	2 AV	Yes	Nil	Good	Yes Varied*	No	WBCT20, fibrinogen	No difference but no outcomes and variable dosing
Otero-Patino, 2012	38/34	*B. asper*	2 AV	Yes	Nil	Good	Yes Varied*	No	WBCT 20, fibrinogen	No difference
Pardal, 2004	38/36	*Bothrops Lachesis*	2 AV	Yes	Nil	Good	Unclear	No	WBCT20, fibrinogen, D-dimer,	No difference
Warrell, 1974	23/23	*E. ocellatus*	2 AV	No	Nil	Nil	Yes Varied*	No	WBCT20, fibrinogen	Unclear difference in outcomes. Dose and AV confounded.
Warrell, 1980	7/7	*E. ocellatus*	2 AV	No	Nil	Nil	Yes Varied*	No	WBCT20, fibrinogen, factor II, X, XIII	Study too small for any conclusion
Cardoso, 1993	39/41/41	*B. jararaca*	3 AV	Yes	Nil	Nil	Yes Varied*	No	WBCT20, fibrinogen, D-dimer	Similar effectiveness of all three antivenoms
Otero-Patino, 1998	30/27/22	*Bothrops*	3 AV	Partial	Partial	Nil	Yes Varied*	No	WBCT30, fibrinogen	Similar effectiveness of all three antivenoms
Smalligan, 2007	82/87/41	*Bothrops Lachesis*	3 AV	Yes	Yes	Good	Yes Varied*	Proportion with clotting blood at 6 hr	WBCT 20	No statistically significant difference in the primary outcome or 24-hour outcome
Warrell et al., 1986	15/15/16	*C. rhodostoma*	3 AV	No	Nil	Nil	Yes Varied*	No	WBCT20, fibrinogen	Equally effective based on outcomes of restoration of coagulation
Dart, 2001	16/15	North American Crotalid	2 doses	No	Yes	Good	Yes	Yes	Fibrinogen, PT	Both dosing regimens were equally effective
Jorge, 1995	88/82	*Bothrops jararaca*	4 vs. 2 vials	Yes	Yes	Nil	Yes	No	WBCT10/30, fibrinogen, FDP	Both dosing regimens were equally effective
Karnchanachetanee, 1994	13/11	*D. russelii*	Low vs. high dose	No	Nil	Nil	Yes	No	WBCT20	No difference but no clear information on clotting outcomes
Paul, 2004	50/50	*D. russelii E. carinatus*	6 vs. 12 vials AV	No	Nil	Nil	Yes	No	WBC time, PT	No difference
Thomas and Jacob, 1985	26/27	Probably *E. carinatus D. russelii*	High vs. low dose	No	Nil	Nil	Yes	No	WBCT15	No difference. Unusual dosing regimens.
Myint-Lwin, 1989	14/14	*D. russelii*	AV vs. heparin+AV	No	Nil	Nil	Yes	No	fibrinogen, factor V, X	No difference with the addition of heparin
Paul, 2003	57/65	*D. russelii*	AV vs. heparin+AV	No	Nil	Nil	Yes	No	WBCT30, PT, fibrinogen	No difference with the addition of heparin
Paul, 2007	40/40	Probably *D. russelii E. carinatus*	AV vs. deltaparin+AV	No	Nil	Nil	Yes	No	WBCT30, PT, fibrinogen	No difference with the addition of deltaparin
Shah, 1986	25/25	*E. carinatus*	AV vs. heparin+AV	No	Nil	Nil	Yes	No	Undefined clotting time, PT, fibrinogen	More rapid improvement in haematological parameters
Warrell, 1976	7/7	*E. carinatus*	AV vs. heparin+AV	No	Nil	Nil	Yes	No	WBCT20, factor V, VIII, II	No difference with the addition of heparin
Sellahewa, 1994	8/7	Probably *D. russelii E. carinatus*	AV vs. IVIG+AV	No	Partial	Nil	Yes	No	WBCT12	No statistically significant differences and patients were given further antivenom

* Varied based on the clinical assessment of the severity on admission;

† May differ from the author's conclusion, see text. Abbreviations: AV – antivenom; WBCT20 – 20-minute whole blood clotting test (or 12-, 15-, or 30-minute); WBC time – whole blood clotting time; IVIG – intravenous immunoglobulin; PT – prothrombin time;

**Table 3:** Summary of the non-randomised studies if VICC comparing two groups.

Authors	Type of study	N	Snake species	Study Arms	Primary Outcome	Design Problems	VICC measures	Study conclusions†
Bregani, 2006	Prospective (comparison over time)	130/98/60	*E. ocellatus*	3 AV	No	Multiple outcomes without correction for multiple testing	WBCT30	Sii Polyvalent is not effective compared to two other antivenoms.
Warrell, 1977	Observational	48/65/4	*E. ocellatus*	3 AV	No	Unbalanced groups, patients re-treated with different antivenom	WBCT20	Suggests that one antivenom was inferior.
Abubakar, 2010	Dose finding study	24	*E. ocellatus*	3 AV	Yes	Nil major	WBCT20	Established the dose for two antivenoms for a clinical trial
Visser, 2008	Prospective	278(48)/66	*E. ocellatus*	2 AV	No	Only 114 had WBCT20 done, and not all positive. Multiple outcomes.	WBCT20	One antivenom was superior to the other based on death rate and antivenom dose
Isbister, 2009	Prospective cohort	112/29	Australian elapids	Early (<6 h) vs. late (>6 h) AV; [AV vs AV+FFP]	Yes	Primary analysis was a time to event analysis, secondary analysis compared late vs. early	INR	No difference between early and late AV group on VICC recovery. More rapid recovery with FFP.
Suchithra, 2008	Prospective cohort	142/127 [102/25]	Probably *D. russelii, E. carinatus*	Early (<6 h) vs. late (>6 h) AV	No	Subgroup analysis. Error in comparison of early and late AV for WBCT – not parametric and not significant (p = 0.15)	WBCT20, PT, aPTT	No difference between early and late AV. However, incorrect statistical analysis may have missed significant difference for WBCT20.
Trevett, 1995	Prospective observational study	33/31	*O. scutellatus*	Early (<4 h) vs. late (>4 h) AV	No	Small subgroup analysis.	WBCT20	No difference between early and late AV in time to recovery of WBCT20.
Mann, 1978	Retrospective study	9/9	*E. coloratus*	AV vs. no AV	No	Sample size too small.	Fibrinogen	No difference in recovery of fibrinogen with AV.
Mion, 2013	Prospective observational study (47 vs. 13)	47/13	*E. pyramidum*	AV vs. no AV	No	Multiple outcomes	FibrinogenaPTT, PT	Significant difference in recovery of all coagulation parameters with AV compared to no AV.
Brown, 2009	Retrospective/Prospective study (106 vs. 21)	106/21	Australian elapids	AV vs. no AV; FFP vs. no FFP	Yes	Two separate studies amalgamated.	INR, aPTT, Fibrinogen	FFP given within 4 hours of antivenom is associated with a more rapid recovery of the INR.
Win-Aung, 1996	Prospective, observational study	34/82	*D. siamensis*	IM AV vs IV AV	No	IM groups less severely envenomed, multiple outcomes and unclear if all patients given IM included	WBCT20	Authors report a significant difference (p = 0.03) but poor design and unbalanced groups suggest this may not be a significant difference.
Srimannarayana, 2004	Prospective with two arms randomised	30/30/30	Probably *D. russelii, E. carinatus*	Three dose levels	No	Randomised controlled trial but included one non-randomised arm	WBCT30	No difference
Tin, 1992	Prospective two arms	10/10	*D. russeli*	AV vs. heparin+AV	No	Small study with multiple outcomes	Fibrinogenfactor V, X	No difference when adding heparin

† May differ to the author's conclusion, see text. Abbreviations: AV – antivenom; WBCT20 – 20 minute whole blood clotting test (or 12, 15 or 30 minute); PT – prothrombin time; aPTT – activated partial thromboplastin time; INR – international normalised ratio; IM – intramuscular; IV - intravenous.

Unfortunately there were major design issues with most of the randomised controlled trials, including lack of definition of a primary outcome or post-hoc definition of the primary outcome, no information on allocation concealment, no information on randomisation, no information on antivenom dose, or varying doses given to patients, and all but two studies [48], [66] were underpowered with no sample size calculation (Table 2). The primary assessment of coagulopathy in these studies varied with different whole blood clotting tests and times (12, 15, 20, or 30 minutes) and measurement of the PT, aPTT, fibrin degradation products (FDP), D-Dimer, or fibrinogen, making comparison between studies difficult and reliability of the WBCT outcomes questionable. Many of the studies used a restoration of “coagulable blood” based on the WBCT as the major outcome, which is problematic because the reliability of WBCT20 for VICC has been recently questioned [16].

The greatest limitation of the randomised controlled trials was the absence of placebo controlled trials, so none of the trials could effectively address the question as to whether antivenom was beneficial in treating VICC. In nine of 14 studies, the authors concluded equal effectiveness of two or three antivenoms, and four of five studies of different doses or dosing regimens concluded equal effectiveness. The commonest interpretation of these studies is that antivenoms are equally effective. However, these studies actually provide no evidence for antivenom effectiveness and can be interpreted as two antivenoms being equally ineffective. All that can be concluded from these studies is that using any one of the two or three antivenoms is similarly effective.

In five of the 14 studies comparing different antivenoms, the authors concluded that one antivenom was superior to the other(s). However, on reviewing these studies, there were problems with study design or dose was confounded with antivenom type (i.e., the antivenom was less effective because an insufficient dose was given [49], [68], [69]). One of the better clinical trials of antivenom for VICC was the randomised comparative trial of EchiTAb Plus equine antivenom and EchiTAb G ovine antivenom for *Echis ocellatus* envenoming, which concluded that EchiTAb Plus was slightly more effective than the other [48]. However, this study was designed as a non-inferiority study, and therefore the authors can only conclude that neither antivenom was inferior to the other, based on the primary outcome. It is incorrect to then use a one-sided p-value to suggest that one antivenom was superior to the other. Any conclusion on positive secondary outcomes is also questionable in a non-inferiority trial design. The study by Ariaranee et al., in 2001, concluded that Haffkine antivenom was more effective than Polonga TAb and that a larger dose of Polonga TAb was required [49]. They based this on only 74% of patients having coagulable blood 6 hours after Haffkine antivenom compared to 41% after Polonga Tab. However, 12 hours after antivenom, 95% of patients receiving Haffkine antivenom had coagulable blood compared to 86% receiving Polonga Tab, which was unlikely to be significant. The authors did not define a primary outcome, so the study can be interpreted differently based on whether a 6-hour or 12-hour outcome is used. The study by Smalligan et al. also concluded that one antivenom was more effective, but the study was underpowered (210 recruited versus 300 required for the sample size) and the result was not significant (p = 0.054) for the primary outcome at 6 hours if only patients given the standard initial dose were included (the outcome that the sample size appeared to be based on) [66]. Warrell et al., in 1974, compared S.A.I.M.R. antivenom and Behringwerke antivenom, but their outcomes included the dose of antivenom required and the proportion with coagulation restored. The study showed that larger amounts of Behringwerke antivenom were required, and not necessarily that it was less effective [68]. Warrell et al., in 1980, conclude in another underpowered study of 14 patients with no clearly defined primary outcome that Behringwerke antivenom was unreliable [69]. The trial was too small to show any significant difference between the two antivenoms.

In contrast to the randomised controlled trials, some non-randomised comparative studies do provide evidence for and against the effectiveness of antivenom for VICC. A number of Australian studies and one study of Papuan taipan bites found that antivenom does not prevent or speed the recovery of VICC in Australian elapid envenoming [3], [9]. This was supported by computer modelling of the coagulation pathway that showed that antivenom needed to be given almost immediately to prevent VICC in Papuan taipan and Australian elapids [44], [45]. One Australian study found that early (<6 hours after the bite) and late (>6 hours after the bite) administration of antivenom resulted in the same recovery rate of VICC with 3% and 33% recovering to an INR of 2 or less after 6 and 12 hours for early antivenom, compared to 3% and 27% for late antivenom [9]. Trevett et al. also showed that early antivenom (<4 hours) versus late antivenom (>4 hours) in Papuan taipan did not result in a more rapid recovery of the coagulopathy [75]. These studies suggest that there is a limited role for antivenom in the treatment of VICC resulting from Australasian elapid envenoming. However, other studies have shown that antivenom can prevent other clinical effects of envenoming such as neurotoxicity and myotoxicity [75], [80], so evidence of VICC and therefore envenoming remains an indication for antivenom. However, in brown snake (*Pseudonaja*) envenoming, where the major clinical syndrome is VICC [81], it could be argued that antivenom does not improve outcomes, and it might be ethical to undertake a placebo controlled trial of antivenom.

Different to Australian antivenom, studies of *Echis* species have demonstrated an important role for antivenom in the treatment of VICC, because antivenom greatly shortens the duration of the coagulopathy. A recent study of *Echis* envenoming by Mion et al. showed that there was a much more rapid recovery of the PT, aPTT, and fibrinogen levels in patients given antivenom compared to those not treated [22]. The mean recovery times to fibrinogen >1 g/l was 7.5 days versus 40 hours; to a PT>50% was 5.8 days versus 25 hours, and to an aPTT<1.5 times normal was 4.7 days versus 9 hours, for untreated and antivenom treated patients respectively [22]. This supports earlier work that found the mortality from *Echis* envenoming was reduced in patients treated with specific antivenoms in Nigeria and the time to the restoration of clotting was much more rapid [8]. The study by Visser et al. reports an increased mortality for patients envenomed by *E. ocellatus* given Asna Antivenom C (Bharat Serum and Vaccines Ltd.) compared to FAV-Afrique (Aventis-Pasteur) and significantly more doses required until the WBCT20 normalised [76]. The failure of Indian antivenom is likely due to the fact that a different *Echis* spp. is used to produce it. There is therefore sufficient evidence from non-randomised studies that doing a placebo controlled trial would be considered unethical.

One study investigated whether the time of antivenom post-bite affected the time to recovery of the coagulopathy in Russell's viper and carpet vipers (*E. carinatus*) in India [74]. The study reported that early antivenom (<6 hours after bite) compared to late antivenom (>6 hours) resulted in a more rapid recovery of the WBCT20, but not the time to recovery of standard coagulation studies (INR, aPTT). This result is difficult to interpret because it included two snake types with different types of procoagulant toxins and did not clearly define outcomes a priori. In addition, there is an error in the statistical analysis because comparison of the values reported in Table 4 in [74] does not give a significant difference in recovery of the WBCT20 between early and late antivenom administration.

The failure of antivenom for VICC in Australia and success of antivenom for VICC from *Echis* spp. in Africa demonstrates that studies of one snake (and therefore one procoagulant toxin) cannot be generalised to other snakes. Studies are required for each major group of snakes or toxins in different parts of the world, although understanding the mechanisms of the procoagulant toxins should inform empirical studies of different antivenoms. The prothrombin activators in Australasian elapids (Group C and D) [4] are similar to the prothrombinase complex in humans and therefore likely to be removed rapidly by pathways that eliminate human prothrombinase. However, the prothrombin activators in the venoms of *Echis* spp. (Group A and B) are metalloproteinases, which differ from the human clotting factors, and therefore are unlikely to be removed by normal elimination pathways.

The study by Win-Aung et al. reported the effectiveness of intramuscular antivenom and is the only study of intramuscular antivenom [77]. This finding is not consistent with pharmacokinetic studies of intramuscular antivenom, which suggest very slow and delayed absorption [82]. The study by Win-Aung did not have an appropriate control group, which was clearly shown by the fact that “test” patients had significantly lower venom concentrations (p<0.001) than the control patients and so were more mildly envenomed. Considering the statistical significance in the number of patients with coagulopathy between groups was p = 0.03, it is likely that this is accounted for by the “test” group being less severely envenomed. Another concern with the study is that the authors do not report how many patients actually got intramuscular antivenom; they only included those cases in which venom was detected in blood [77]. Contrary to the authors' conclusions this study does not support the effectiveness of intramuscular antivenom, and this route of administration should not be used.

The remaining non-randomised studies of antivenom for VICC were of poor quality (Table 3). Bregani et al. undertook a study of three different antivenoms for *Echis* in Africa and suggested that one was ineffective and was associated with a higher mortality and slower return of clotting function [20]. However, the study compared three groups and multiple outcomes without correcting for this, so not all of these differences may have been significant. Mann et al. undertook a small retrospective study of no antivenom versus antivenom for envenoming by *E. colorata*. Although it suggests the recovery in fibrinogen was similar, the study was small and there was no clear definition of recovery [73]. Two studies not included in the 15 simply compared the occurrence of coagulation abnormalities in the envenomed patients and not the recovery of coagulation [23], [83]. This meant that severity of envenoming was confounded with antivenom treatment, making it difficult to assess the effect of antivenom.

Another important issue is determining the effective dose of antivenom. There has been significant contention in Australia regarding the dose of antivenom. Recent studies have demonstrated that one vial of antivenom is as effective as two or more vials of antivenom for VICC resulting from snake envenoming [81]. However, it is clear from many of the studies that some antivenoms are not effective because an insufficient dose of the antivenom has been given [49], [76]. It is essential that future studies do not confound dose and type of antivenom, and that the optimal dose of antivenom is determined in pre-clinical studies or small dose-finding studies prior to larger controlled trials of antivenom effectiveness.

There are numerous observational studies that report the effectiveness of antivenom, but they simply report the restoration of coagulability for a single group of patients that are all given antivenom. Clearly, further studies of antivenom for VICC are required, but conducting placebo controlled trials is challenging if not impossible due to ethical issues. Good observational studies and historical control studies will hopefully help provide better evidence for the role of antivenom in VICC from different snakes.

## Other Treatments

### Clotting factor replacement

VICC is characterised by low or undetectable levels of one or more clotting factors, most commonly fibrinogen. Antivenom will only stop the consumptive process so once it has been given it still takes 24 to 48 hours for full recovery of the clotting factors [3]. While the clotting factors are being re-synthesized by the liver there is a period of time during which the patient remains at risk of haemorrhage. For this reason, clotting factor replacement has been suggested as an adjunct treatment for VICC. The most commonly used factor replacement is fresh frozen plasma because it is the most widely available and contains almost all the important factors, such as fibrinogen, factor V, factor VIII, and factor X.

Clotting factor replacement for VICC is controversial because of the concern that it may worsen VICC by providing more clotting factors (substrate) for the procoagulant toxins [84], [85]. However, it has been assumed that once antivenom has been given and the toxins are bound, clotting factor replacement is likely to speed the rate of recovering. Two observational studies from Australia support this [9], [21] but a more recent randomised controlled trial in Australia only partly supports the use of fresh frozen plasma (FFP) and raises concern about the early use of FFP [86].

The recent randomised controlled trial of FFP for treating VICC in Australian snake envenoming shows that the administration of FFP within 4 hours of antivenom results in more rapid restoration of clotting function in the majority of patients [86]. In a study of 65 patients, 30 of 41 patients (73%) randomised to FFP had an INR of <2 six hours after antivenom compared to only six of 24 patients (25%) not given FFP (absolute difference 48%; 95% confidence interval (CI): 23%–73%; p = 0.0002). However, there was no difference in time to discharge and the study was too small to detect any different in major haemorrhage between FFP and no FFP. An interesting finding of the study was that non-responders in the FFP arm were given FFP significantly earlier post-bite (not post-antivenom) than those who responded to FFP (4.7 hours versus 7.3 hours; p = 0.002) [86]. The reason for this finding is not completely clear, but clotting factor studies done in a subgroup of patients demonstrated that those receiving early FFP had evidence of consumption after the FFP was given with increasing D-Dimer and decreasing fibrinogen. However, all these patients had received antivenom prior to the FFP, suggesting that the active clotting factors were endogenous ones activated in the initial consumptive process and not the procoagulant toxin.

Reactions to FFP are well recognised, but are relatively uncommon [87]. There were no adverse reactions in the randomised controlled trial by Isbister et al. in 2013 that could be directly attributed to the FFP. However, the study was small and uncommon complications such as transfusion-related acute lung injury (TRALI) and anaphylaxis must be considered when balancing the risk of FFP versus the benefit. The current evidence would suggest that FFP should be administered in patients with acute bleeding and is more likely to be effective if given more than 6 hours after the bite. However, there is much less evidence to support FFP in patients with VICC without active bleeding, and larger studies are required to better define this patient group. Nevertheless, in life-threatening bleeding from VICC, such as with intracranial haemorrhage, delaying the use of FFP based on these findings is not recommended.

There is far less information on other forms of factor replacement, including cryoprecipitate, prothrombinex, or single factor concentrations. It would seem theoretically useful to give cryoprecipitate to patients bitten by snakes with thrombin-like enzymes, who mainly have a fibrinogen deficiency. However, there is little evidence to support this and patients only need low levels of fibrinogen to have close-to-normal clotting function. One retrospective study included patients given cryoprecipitate, but the numbers were too small for any analysis [21].

### Heparin

Heparin has been suggested for the treatment of VICC resulting from viper envenoming, but its use is controversial and there is little evidence to support its effectiveness. Three of five randomised comparison studies concluded that heparin and antivenom are more effective than antivenom alone [24], [53], [61], [63], [65]. There was one non-randomised study that concluded no benefit [78]. However, all these studies were poorly designed with poor definition of primary outcomes, blinded allocation, and clotting tests.

Shah et al. reported in 1986 that the addition of heparin resulted in a great proportion of patients with normalised haematological parameters of four different time points compared to antivenom alone [65]. This result needs to be interpreted with caution because it is unclear which parameters had to normalise and multiple time points were used [65]. Paul et al reported two studies investigating the effect of heparin that showed no significant difference despite the conclusion heparin was effective. The first study showed no statistical benefit of the addition of heparin, including no effect on the clotting times, which were the same between antivenom alone and antivenom and heparin [61]. The authors incorrectly suggest that heparin resulted in an improved morbidity and mortality, based on very small and non-significant differences in some outcomes [61]. In the second, smaller study the authors found no significant difference between the antivenom and antivenom with heparin on all outcomes [63]. The three trials that concluded heparin was not effective were all too small (N = 14, 20, 28) to detect any but the largest difference between treatments, due to type II errors. In addition, all these studies had poorly defined outcomes, including no pre-specified primary outcome [24], [53], [78]. There is insufficient evidence to support the use of heparin, but well-designed, large studies are required to confirm that there is no effect.

## Conclusions

VICC is one of the most important clinical syndromes that occurs with snake envenoming and it includes a broad range of factor deficiencies depending on the particular procoagulant toxin in the snake venom. Diagnosis and monitoring of the coagulopathy is problematic, particularly in resource-poor countries where the only clotting test available is the whole blood clotting test. Research is required to develop more reliable cheap clotting tests to be used for the diagnosis and treatment of VICC. Antivenom is the major treatment for VICC, but there is little high-quality evidence to support its effectiveness. Observational studies have suggested that it may be highly effective for some snakes (e.g., *Echis* spp.) and ineffective for other snakes (e.g., Australasian elapids). Antivenom is not risk free and adverse reactions can be quite common and potentially severe. There is evidence to support the use of FFP in bleeding patients with VICC. There is no evidence to support the use of heparin. In all cases it is important to observe for signs of external and internal bleeding. Patients should be observed in hospital until clotting function has normalised.

**Key Learning Points**

- Venom-induced consumption coagulopathy (VICC) results from the activation of the clotting pathway by procoagulant snake toxins and consumption of clotting factors.
- Investigation of VICC requires laboratory-based clotting studies until accurate and cheap bedside tests are available.
- There are no placebo controlled trials of antivenom, and effectiveness is not supported by numerous clinical trials comparing antivenoms.
- Non-randomised observational studies with control groups suggest that antivenom may be effective for some snakes but not others.
- There is little evidence to support the use of heparin, and fresh frozen plasma is likely to be beneficial only in actively bleeding patients.

**Top Five Papers**

1. Isbister GK, Scorgie FE, O'Leary MA, Seldon M, Brown SG, et al. (2010) Factor deficiencies in venom-induced consumption coagulopathy resulting from Australian elapid envenomation: Australian Snakebite Project (ASP-10). J Thromb Haemost 8: 2504–2513.
2. Isbister GK (2009) Procoagulant snake toxins: laboratory studies, diagnosis, and understanding snakebite coagulopathy. Semin Thromb Hemost 35: 93–103.
3. Mion G, Larreche S, Benois A, Petitjeans F, Puidupin M (2013) Hemostasis dynamics during coagulopathy resulting from Echis envenomation. Toxicon 76: 103–109.
4. Isbister GK, Duffull SB, Brown SG (2009) Failure of antivenom to improve recovery in Australian snakebite coagulopathy. QJM 102: 563–568.
5. Abubakar IS, Abubakar SB, Habib AG, Nasidi A, Durfa N, et al. (2010) Randomised controlled double-blind non-inferiority trial of two antivenoms for saw-scaled or carpet viper (Echis ocellatus) envenoming in Nigeria. PLoS Negl Trop Dis 4: e767.

## References

[1] Kasturiratne A, Wickremasinghe AR, de Silva N, Gunawardena NK, Pathmeswaran A, et al (2008) The global burden of snakebite: a literature analysis and modelling based on regional estimates of envenoming and deaths. PLoS Med 5: e218. 18986210 10.1371/journal.pmed.0050218 PMC2577696

[2] Isbister GK (2010) Snakebite doesn't cause disseminated intravascular coagulation: coagulopathy and thrombotic microangiopathy in snake envenoming. Semin Thromb Hemost 36: 444–451. 20614396 10.1055/s-0030-1254053

[3] Isbister GK, Scorgie FE, O'Leary MA, Seldon M, Brown SG, et al (2010) Factor deficiencies in venom-induced consumption coagulopathy resulting from Australian elapid envenomation: Australian Snakebite Project (ASP-10). J Thromb Haemost 8: 2504–2513. 20831619 10.1111/j.1538-7836.2010.04050.x

[4] Isbister GK (2009) Procoagulant snake toxins: laboratory studies, diagnosis, and understanding snakebite coagulopathy. Semin Thromb Hemost 35: 93–103. 19308897 10.1055/s-0029-1214152

[5] Aitchison JM (1990) Boomslang bite–diagnosis and management. A report of 2 cases. S Afr Med J 78: 39–42. 2363083

[6] Lu Q, Clemetson JM, Clemetson KJ (2005) Snake venoms and hemostasis. J Thromb Haemost 3: 1791–1799. 16102046 10.1111/j.1538-7836.2005.01358.x

[7] Phillips RE, Theakston RD, Warrell DA, Galigedara Y, Abeysekera DT, et al (1988) Paralysis, rhabdomyolysis and haemolysis caused by bites of Russell's viper (Vipera russelli pulchella) in Sri Lanka: failure of Indian (Haffkine) antivenom. Q J Med 68: 691–715. 3256900

[8] Warrell DA, Davidson N, Greenwood BM, Ormerod LD, Pope HM, et al (1977) Poisoning by bites of the saw-scaled or carpet viper (Echis carinatus) in Nigeria. Q J Med 46: 33–62. 866568

[9] Isbister GK, Duffull SB, Brown SG (2009) Failure of antivenom to improve recovery in Australian snakebite coagulopathy. QJM 102: 563–568. 19570990 10.1093/qjmed/hcp081

[10] Kularatne SA, Budagoda BD, Gawarammana IB, Kularatne WK (2009) Epidemiology, clinical profile and management issues of cobra (Naja naja) bites in Sri Lanka: first authenticated case series. Trans R Soc Trop Med Hyg 103: 924–930. 19439335 10.1016/j.trstmh.2009.04.002

[11] Kularatne SA, Sivansuthan S, Medagedara SC, Maduwage K, de Silva A (2011) Revisiting saw-scaled viper (Echis carinatus) bites in the Jaffna Peninsula of Sri Lanka: distribution, epidemiology and clinical manifestations. Trans R Soc Trop Med Hyg 105: 591–597. 21868049 10.1016/j.trstmh.2011.07.010

[12] Maduwage K, Kularatne K, Wazil A, Gawarammana I (2011) Coagulopthy, acute kidney injury and death following Hypnale zara envenoming: the first case report from Sri Lanka. Toxicon 58: 641–643. 21967813 10.1016/j.toxicon.2011.09.014

[13] Maduwage K, Scorgie FE, Silva A, Shahmy S, Mohamed F, et al (2013) Hump-nosed pit viper (Hypnale hypnale) envenoming causes mild coagulopathy with incomplete clotting factor consumption. Clin Toxicol (Phila) 51: 527–531. 23879180 10.3109/15563650.2013.811589

[14] Ariaratnam CA, Thuraisingam V, Kularatne SA, Sheriff MH, Theakston RD, et al (2008) Frequent and potentially fatal envenoming by hump-nosed pit vipers (Hypnale hypnale and H. nepa) in Sri Lanka: lack of effective antivenom. Trans R Soc Trop Med Hyg 102: 1120–1126. 18455743 10.1016/j.trstmh.2008.03.023

[15] Sano-Martins IS, Fan HW, Castro SC, Tomy SC, Franca FO, et al (1994) Reliability of the simple 20 minute whole blood clotting test (WBCT20) as an indicator of low plasma fibrinogen concentration in patients envenomed by Bothrops snakes. Butantan Institute Antivenom Study Group. Toxicon 32: 1045–1050. 7801340 10.1016/0041-0101(94)90388-3

[16] Isbister GK, Maduwage K, Shahmy S, Mohamed F, Abeysinghe C, et al (2013) Diagnostic 20-min whole blood clotting test in Russell's viper envenoming delays antivenom administration. QJM 106: 925–932. 23674721 10.1093/qjmed/hct102

[17] Daudu I, Theakston RD (1988) Preliminary trial of a new polyspecific antivenom in Nigeria. Ann Trop Med Parasitol 82: 311–313. 3250345 10.1080/00034983.1988.11812249

[18] Chippaux JP, Lang J, Eddine SA, Fagot P, Rage V, et al (1998) Clinical safety of a polyvalent F(ab′)2 equine antivenom in 223 African snake envenomations: a field trial in Cameroon. VAO (Venin Afrique de l'Ouest) Investigators. Trans R Soc Trop Med Hyg 92: 657–662. 10326114 10.1016/s0035-9203(98)90802-1

[19] Chippaux JP, Lang J, Amadi-Eddine S, Fagot P, Le Mener V (1999) Short report: treatment of snake envenomations by a new polyvalent antivenom composed of highly purified F(ab)2: results of a clinical trial in northern Cameroon. Am J Trop Med Hyg 61: 1017–1018. 10674688 10.4269/ajtmh.1999.61.1017

[20] Bregani E, Cantoni F, Tantardini F (2006) Snake bites in South Chad. Comparision between three different polyvalent anti-snake venom immunotherapies. Giornale Italiano Di Medicina Tropicale 11: 25–28.

[21] Brown SG, Caruso N, Borland ML, McCoubrie DL, Celenza A, et al (2009) Clotting factor replacement and recovery from snake venom-induced consumptive coagulopathy. Intensive Care Med 35: 1532–1538. 19547954 10.1007/s00134-009-1556-7

[22] Mion G, Larreche S, Benois A, Petitjeans F, Puidupin M (2013) Hemostasis dynamics during coagulopathy resulting from Echis envenomation. Toxicon 76: 103–109. 24070638 10.1016/j.toxicon.2013.09.003

[23] Sotelo N (2008) Review of treatment and complications in 79 children with rattlesnake bite. Clin Pediatr (Phila) 47: 483–489. 18192639 10.1177/0009922807311734

[24] Warrell DA, Pope HM, Prentice CR (1976) Disseminated intravascular coagulation caused by the carpet viper (Echis carinatus): trial of heparin. Br J Haematol 33: 335–342. 1276079 10.1111/j.1365-2141.1976.tb03549.x

[25] Kamiguti AS, Rugman FP, Theakston RD, Franca FO, Ishii H, et al (1992) The role of venom haemorrhagin in spontaneous bleeding in Bothrops jararaca envenoming. Butantan Institute Antivenom Study Group. Thromb Haemost 67: 484–488. 1631797

[26] Antonypillai CN, Wass JA, Warrell DA, Rajaratnam HN (2011) Hypopituitarism following envenoming by Russell's vipers (Daboia siamensis and D. russelii) resembling Sheehan's syndrome: first case report from Sri Lanka, a review of the literature and recommendations for endocrine management. QJM 104: 97–108. 21115460 10.1093/qjmed/hcq214

[27] Jeevagan V, Katulanda P, Gnanathasan CA, Warrell DA (2013) Acute pituitary insufficiency and hypokalaemia following envenoming by Russell's viper (Daboia russelii) in Sri Lanka: Exploring the pathophysiological mechanisms. Toxicon 63: 78–82. 23212048 10.1016/j.toxicon.2012.11.012

[28] Offerman SR, Barry JD, Schneir A, Clark RF (2003) Biphasic rattlesnake venom-induced thrombocytopenia. J Emerg Med 24: 289–293. 12676300 10.1016/s0736-4679(02)00763-1

[29] Odeleye AA, Presley AE, Passwater ME, Mintz PD (2004) Report of two cases: Rattlesnake venom-induced thrombocytopenia. Ann Clin Lab Sci 34: 467–470. 15648790

[30] Johnston CI, Brown SG, O'Leary MA, Currie BJ, Greenberg R, et al (2013) Mulga snake (Pseudechis australis) envenoming: a spectrum of myotoxicity, anticoagulant coagulopathy, haemolysis and the role of early antivenom therapy - Australian Snakebite Project (ASP-19). Clin Toxicol (Phila) 51: 417–424. 23586640 10.3109/15563650.2013.787535

[31] Warrell DA, editor (1992) The global problem of snake bite: its prevention and treatments. Singapore: National University of Singapore.

[32] Gutierrez JM, Leon G, Lomonte B (2003) Pharmacokinetic-pharmacodynamic relationships of immunoglobulin therapy for envenomation. Clin Pharmacokinet 42: 721–741. 12846594 10.2165/00003088-200342080-00002

[33] Theakston RD, Warrell DA (1991) Antivenoms: a list of hyperimmune sera currently available for the treatment of envenoming by bites and stings. Toxicon 29: 1419–1470. 1801323 10.1016/0041-0101(91)90002-9

[34] Heard K, O'Malley GF, Dart RC (1999) Antivenom therapy in the Americas. Drugs 58: 5–15. 10439926 10.2165/00003495-199958010-00002

[35] Isbister GK (2010) Antivenom efficacy or effectiveness: the Australian experience. Toxicology 268: 148–154. 19782716 10.1016/j.tox.2009.09.013

[36] Riviere G, Choumet V, Audebert F, Sabouraud A, Debray M, et al (1997) Effect of antivenom on venom pharmacokinetics in experimentally envenomed rabbits: toward an optimization of antivenom therapy. J Pharmacol Exp Ther 281: 1–8. 9103473

[37] Pepin-Covatta S, Lutsch C, Grandgeorge M, Lang J, Scherrmann JM (1996) Immunoreactivity and pharmacokinetics of horse anti-scorpion venom F(ab′)2-scorpion venom interactions. Toxicol Appl Pharmacol 141: 272–277. 8917700 10.1006/taap.1996.0284

[38] Pepin-Covatta S, Lutsch C, Lang J, Scherrmann JM (1998) Preclinical assessment of immunoreactivity of a new purified equine F(ab′)2 against European viper venom. J Pharm Sci 87: 221–225. 9519157 10.1021/js9701824

[39] Isbister GK, Woods D, Alley S, O'Leary MA, Seldon M, et al (2010) Endogenous thrombin potential as a novel method for the characterization of procoagulant snake venoms and the efficacy of antivenom. Toxicon 56: 75–85. 20338189 10.1016/j.toxicon.2010.03.013

[40] Isbister GK, O'Leary MA, Schneider JJ, Brown SG, Currie BJ (2007) Efficacy of antivenom against the procoagulant effect of Australian brown snake (Pseudonaja sp.) venom: in vivo and in vitro studies. Toxicon 49: 57–67. 17055016 10.1016/j.toxicon.2006.09.007

[41] Ramos-Cerrillo B, de Roodt AR, Chippaux JP, Olguin L, Casasola A, et al (2008) Characterization of a new polyvalent antivenom (Antivipmyn Africa) against African vipers and elapids. Toxicon 52: 881–888. 18926842 10.1016/j.toxicon.2008.09.002

[42] Segura A, Villalta M, Herrera M, Leon G, Harrison R, et al (2010) Preclinical assessment of the efficacy of a new antivenom (EchiTAb-Plus-ICP) for the treatment of viper envenoming in sub-Saharan Africa. Toxicon 55: 369–374. 19699756 10.1016/j.toxicon.2009.08.010

[43] Bogarin G, Morais JF, Yamaguchi IK, Stephano MA, Marcelino JR, et al (2000) Neutralization of crotaline snake venoms from Central and South America by antivenoms produced in Brazil and Costa Rica. Toxicon 38: 1429–1441. 10758277 10.1016/s0041-0101(99)00236-6

[44] Tanos PP, Isbister GK, Lalloo DG, Kirkpatrick CM, Duffull SB (2008) A model for venom-induced consumptive coagulopathy in snake bite. Toxicon 52: 769–780. 18831981 10.1016/j.toxicon.2008.08.013

[45] Gulati A, Isbister GK, Duffull SB (2013) Effect of Australian elapid venoms on blood coagulation: Australian Snakebite Project (ASP-17). Toxicon 61: 94–104. 23151381 10.1016/j.toxicon.2012.11.001

[46] Wajima T, Isbister GK, Duffull SB (2009) A comprehensive model for the humoral coagulation network in humans. Clin Pharmacol Ther 86: 290–298. 19516255 10.1038/clpt.2009.87

[47] Stone SF, Isbister GK, Shahmy S, Mohamed F, Abeysinghe C, et al (2013) Immune response to snake envenoming and treatment with antivenom; complement activation, cytokine production and mast cell degranulation. PLoS Negl Trop Dis 7: e2326. 23936562 10.1371/journal.pntd.0002326 PMC3723557

[48] Abubakar IS, Abubakar SB, Habib AG, Nasidi A, Durfa N, et al (2010) Randomised controlled double-blind non-inferiority trial of two antivenoms for saw-scaled or carpet viper (Echis ocellatus) envenoming in Nigeria. PLoS Negl Trop Dis 4: e767. 20668549 10.1371/journal.pntd.0000767 PMC2910709

[49] Ariaratnam CA, Sjostrom L, Raziek Z, Kularatne SA, Arachchi RW, et al (2001) An open, randomized comparative trial of two antivenoms for the treatment of envenoming by Sri Lankan Russell's viper (Daboia russelii russelii). Trans R Soc Trop Med Hyg 95: 74–80. 11280073 10.1016/s0035-9203(01)90339-6

[50] Cardoso JL, Fan HW, Franca FO, Jorge MT, Leite RP, et al (1993) Randomized comparative trial of three antivenoms in the treatment of envenoming by lance-headed vipers (Bothrops jararaca) in Sao Paulo, Brazil. Q J Med 86: 315–325. 8327649

[51] Dart RC, Seifert SA, Boyer LV, Clark RF, Hall E, et al (2001) A randomized multicenter trial of crotalinae polyvalent immune Fab (ovine) antivenom for the treatment for crotaline snakebite in the United States. Arch Intern Med 161: 2030–2036. 11525706 10.1001/archinte.161.16.2030

[52] Meyer WP, Habib AG, Onayade AA, Yakubu A, Smith DC, et al (1997) First clinical experiences with a new ovine Fab Echis ocellatus snake bite antivenom in Nigeria: randomized comparative trial with Institute Pasteur Serum (Ipser) Africa antivenom. Am J Trop Med Hyg 56: 291–300. 9129531 10.4269/ajtmh.1997.56.291

[53] Myint L, Tin Nu S, Myint Aye M, Than T, Thein T, et al (1989) Heparin therapy in Russell's viper bite victims with impending dic (a controlled trial). Southeast Asian J Trop Med Public Health 20: 271–277. 2532790

[54] Karnchanachetanee C, Hanvivatvong O, Mahasandana S (1994) Monospecific antivenin therapy in Russell's viper bite. J Med Assoc Thai 77: 293–297. 7869015

[55] Otero R, Gutierrez JM, Nunez V, Robles A, Estrada R, et al (1996) A randomized double-blind clinical trial of two antivenoms in patients bitten by Bothrops atrox in Colombia. The Regional Group on Antivenom Therapy Research (REGATHER). Trans R Soc Trop Med Hyg 90: 696–700. 9015522 10.1016/s0035-9203(96)90442-3

[56] Otero-Patino R, Cardoso JL, Higashi HG, Nunez V, Diaz A, et al (1998) A randomized, blinded, comparative trial of one pepsin-digested and two whole IgG antivenoms for Bothrops snake bites in Uraba, Colombia. The Regional Group on Antivenom Therapy Research (REGATHER). Am J Trop Med Hyg 58: 183–189. 9580075 10.4269/ajtmh.1998.58.183

[57] Otero R, Gutierrez JM, Rojas G, Nunez V, Diaz A, et al (1999) A randomized blinded clinical trial of two antivenoms, prepared by caprylic acid or ammonium sulphate fractionation of IgG, in Bothrops and Porthidium snake bites in Colombia: correlation between safety and biochemical characteristics of antivenoms. Toxicon 37: 895–908. 10340829 10.1016/s0041-0101(98)00220-7

[58] Otero R, Leon G, Gutierrez JM, Rojas G, Toro MF, et al (2006) Efficacy and safety of two whole IgG polyvalent antivenoms, refined by caprylic acid fractionation with or without beta-propiolactone, in the treatment of Bothrops asper bites in Colombia. Trans R Soc Trop Med Hyg 100: 1173–1182. 16698053 10.1016/j.trstmh.2006.01.006

[59] Otero-Patino R, Segura A, Herrera M, Angulo Y, Leon G, et al (2012) Comparative study of the efficacy and safety of two polyvalent, caprylic acid fractionated [IgG and F(ab′)2] antivenoms, in Bothrops asper bites in Colombia. Toxicon 59: 344–355. 22146491 10.1016/j.toxicon.2011.11.017

[60] Pardal PP, Souza SM, Monteiro MR, Fan HW, Cardoso JL, et al (2004) Clinical trial of two antivenoms for the treatment of Bothrops and Lachesis bites in the north eastern Amazon region of Brazil. Trans R Soc Trop Med Hyg 98: 28–42. 14702836 10.1016/s0035-9203(03)00005-1

[61] Paul V, Prahlad KA, Earali J, Francis S, Lewis F (2003) Trial of heparin in viper bites. J Assoc Physicians India 51: 163–166. 12725259

[62] Paul V, Pratibha S, Prahlad KA, Earali J, Francis S, et al (2004) High-dose anti-snake venom versus low-dose anti-snake venom in the treatment of poisonous snake bites–a critical study. J Assoc Physicians India 52: 14–17. 15633711

[63] Paul V, Pudoor A, Earali J, John B, Anil Kumar CS, et al (2007) Trial of low molecular weight heparin in the treatment of viper bites. J Assoc Physicians India 55: 338–342. 17844693

[64] Sellahewa KH, Kumararatne MP, Dassanayake PB, Wijesundera A (1994) Intravenous immunoglobulin in the treatment of snake bite envenoming: a pilot study. Ceylon Med J 39: 173–175. 7728916

[65] Shah PK, Chittora MD, Shekhawat JS, Khangaroot D, Vyas MM (1986) Role of heparin in the management of snake (Echis carinatus) bite cases. J Assoc Physicians India 34: 621–623. 3793694

[66] Smalligan R, Cole J, Brito N, Laing GD, Mertz BL, et al (2004) Crotaline snake bite in the Ecuadorian Amazon: randomised double blind comparative trial of three South American polyspecific antivenoms. BMJ 329: 1129. 15539665 10.1136/bmj.329.7475.1129 PMC527684

[67] Thomas PP, Jacob J (1985) Randomised trial of antivenom in snake envenomation with prolonged clotting time. Br Med J (Clin Res Ed) 291: 177–178. 10.1136/bmj.291.6489.177 PMC1416407 3926113

[68] Warrell DA, Davidson NM, Omerod LD, Pope HM, Watkins BJ, et al (1974) Bites by the saw-scaled or carpet viper (Echis carinatus): trial of two specific antivenoms. Br Med J 4: 437–440. 4154124 10.1136/bmj.4.5942.437 PMC1612524

[69] Warrell DA, Warrell MJ, Edgar W, Prentice CR, Mathison J (1980) Comparison of Pasteur and Behringwerke antivenoms in envenoming by the carpet viper (Echis carinatus). Br Med J 280: 607–609. 7370603 10.1136/bmj.280.6214.607 PMC1600696

[70] Warrell DA, Looareesuwan S, Theakston RD, Phillips RE, Chanthavanich P, et al (1986) Randomized comparative trial of three monospecific antivenoms for bites by the Malayan pit viper (Calloselasma rhodostoma) in southern Thailand: clinical and laboratory correlations. Am J Trop Med Hyg 35: 1235–1247. 3538922 10.4269/ajtmh.1986.35.1235

[71] Jorge MT, Cardoso JL, Castro SC, Ribeiro L, Franca FO, et al (1995) A randomized ‘blinded’ comparison of two doses of antivenom in the treatment of Bothrops envenoming in Sao Paulo, Brazil. Trans R Soc Trop Med Hyg 89: 111–114. 7747293 10.1016/0035-9203(95)90678-9

[72] Abubakar SB, Abubakar IS, Habib AG, Nasidi A, Durfa N, et al (2010) Pre-clinical and preliminary dose-finding and safety studies to identify candidate antivenoms for treatment of envenoming by saw-scaled or carpet vipers (Echis ocellatus) in northern Nigeria. Toxicon 55: 719–723. 19874841 10.1016/j.toxicon.2009.10.024

[73] Mann G (1978) Echis colorata bites in Israel: an evaluation of specific antiserum use on the base of 21 cases of snake bite. Toxicol Eur Res 1: 365–369. 754348

[74] Suchithra N, Pappachan JM, Sujathan P (2008) Snakebite envenoming in Kerala, South India: clinical profile and factors involved in adverse outcomes. Emerg Med J 25: 200–204. 18356348 10.1136/emj.2007.051136

[75] Trevett AJ, Lalloo DG, Nwokolo NC, Naraqi S, Kevau IH, et al (1995) The efficacy of antivenom in the treatment of bites by the Papuan taipan (Oxyuranus scutellatus canni). Trans R Soc Trop Med Hyg 89: 322–325. 7660450 10.1016/0035-9203(95)90562-6

[76] Visser LE, Kyei-Faried S, Belcher DW, Geelhoed DW, van Leeuwen JS, et al (2008) Failure of a new antivenom to treat Echis ocellatus snake bite in rural Ghana: the importance of quality surveillance. Trans R Soc Trop Med Hyg 102: 445–450. 18190937 10.1016/j.trstmh.2007.11.006

[77] Win A, Tin T, Khin Maung M, Aye K, Hla P, et al (1996) Clinical trial of intramuscular anti-snake venom administration as a first aid measure in the field in the management of Russell's viper bite patients. Southeast Asian J Trop Med Public Health 27: 494–497. 9185259

[78] Tin Na S, Myint L, Khin Ei H, Tin T, Tun P (1992) Heparin therapy in Russell's viper bite victims with disseminated intravascular coagulation: a controlled trial. Southeast Asian J Trop Med Public Health 23: 282–287. 1345132

[79] Srimannarayana J, Dutta TK, Sahai A, Badrinath S (2004) Rational use of anti-snake venom (ASV): trial of various regimens in hemotoxic snake envenomation. J Assoc Physicians India 52: 788–793. 15909856

[80] Churchman A, O'Leary MA, Buckley NA, Page CB, Tankel A, et al (2010) Clinical effects of red-bellied black snake (Pseudechis porphyriacus) envenoming and correlation with venom concentrations: Australian Snakebite Project (ASP-11). Med J Aust 193: 696–700. 21143062 10.5694/j.1326-5377.2010.tb04108.x

[81] Allen GE, Brown SG, Buckley NA, O'Leary MA, Page CB, et al (2012) Clinical effects and antivenom dosing in brown snake (Pseudonaja spp.) envenoming–Australian snakebite project (ASP-14). PLoS ONE 7: e53188. 23300888 10.1371/journal.pone.0053188 PMC3532501

[82] Isbister GK, O'Leary M, Miller M, Brown SG, Ramasamy S, et al (2008) A comparison of serum antivenom concentrations after intravenous and intramuscular administration of redback (widow) spider antivenom. Br J Clin Pharmacol 65: 139–143. 18171334 10.1111/j.1365-2125.2007.03004.x PMC2291270

[83] Hung DZ, Yu YJ, Hsu CL, Lin TJ (2006) Antivenom treatment and renal dysfunction in Russell's viper snakebite in Taiwan: a case series. Trans R Soc Trop Med Hyg 100: 489–494. 16325876 10.1016/j.trstmh.2005.07.020

[84] Jelinek GA, Smith A, Lynch D, Celenza A, Irving I, et al (2005) The effect of adjunctive fresh frozen plasma administration on coagulation parameters and survival in a canine model of antivenom-treated brown snake envenoming. Anaesth Intensive Care 33: 36–40. 15957689 10.1177/0310057X0503300106

[85] Tibballs J (2005) Fresh frozen plasma after brown snake bite–helpful or harmful? Anaesth Intensive Care 33: 13–15. 15957686 10.1177/0310057X0503300103

[86] Isbister GK, Buckley NA, Page CB, Scorgie FE, Lincz LF, et al (2013) A randomized controlled trial of fresh frozen plasma for treating venom-induced consumption coagulopathy in cases of Australian snakebite (ASP-18). J Thromb Haemost 11: 1310–1318. 23565941 10.1111/jth.12218

[87] Pandey S, Vyas GN (2012) Adverse effects of plasma transfusion. Transfusion 52 Suppl 1: 65S–79S. 22578374 10.1111/j.1537-2995.2012.03663.x PMC3356109

[88] Than T, Hutton RA, Myint L, Khin Ei H, Soe S, et al (1988) Haemostatic disturbances in patients bitten by Russell's viper (Vipera russelli siamensis) in Burma. Br J Haematol 69: 513–520. 3408687 10.1111/j.1365-2141.1988.tb02408.x

[89] Kulapongs P, Boocharngkul S, Tostiarat T (1975) The defibrination syndrome in Malayan pit viper (Agkistrodon rhodostoma). Chiang Mai Medical Bulletin 14: 20.

[90] Hutton RA, Looareesuwan S, Ho M, Silamut K, Chanthavanich P, et al (1990) Arboreal green pit vipers (genus Trimeresurus) of South-East Asia: bites by T. albolabris and T. macrops in Thailand and a review of the literature. Trans R Soc Trop Med Hyg 84: 866–874. 2096527 10.1016/0035-9203(90)90111-q

[91] Rojnuckarin P, Intragumtornchai T, Sattapiboon R, Muanpasitporn C, Pakmanee N, et al (1999) The effects of green pit viper (Trimeresurus albolabris and Trimeresurus macrops) venom on the fibrinolytic system in human. Toxicon 37: 743–755. 10219986 10.1016/s0041-0101(98)00214-1

[92] Li QB, Huang GW, Kinjoh K, Nakamura M, Kosugi T (2001) Hematological studies on DIC-like findings observed in patients with snakebite in south China. Toxicon 39: 943–948. 11223082 10.1016/s0041-0101(00)00232-4

[93] Mori K, Hisa S, Suzuki S, Sugai K, Sakai H, et al (1983) [A case of severe defibrination syndrome due to snake (Rhabdophis tigrinus) bite]. Rinsho Ketsueki 24: 256–262. 6887532

[94] Lalloo DG, Trevett AJ, Owens D, Minei J, Naraqi S, et al (1995) Coagulopathy following bites by the Papuan taipan (Oxyuranus scutellatus canni). Blood Coagulation & Fibrinolysis 6: 65–72. 7540879 10.1097/00001721-199502000-00011

[95] Barrantes A, Solis V, Bolanos R (1985) [Alterations in the coagulation mechanisms of patients bitten by Bothrops asper (Terciopelo)]. Toxicon 23: 399–407. 4024146 10.1016/0041-0101(85)90024-8

[96] Kamiguti AS, Matsunaga S, Spir M, Sano-Martins IS, Nahas L (1986) Alterations of the blood coagulation system after accidental human inoculation by Bothrops jararaca venom. Braz J Med Biol Res 19: 199–204. 3103794

[97] Sano-Martins IS, Tomy SC, Campolina D, Dias MB, de Castro SC, et al (2001) Coagulopathy following lethal and non-lethal envenoming of humans by the South American rattlesnake (Crotalus durissus) in Brazil. QJM 94: 551–559. 11588214 10.1093/qjmed/94.10.551

[98] Kamiguti AS, Cardoso JL (1989) Haemostatic changes caused by the venoms of South American snakes. Toxicon 27: 955–963. 2678605 10.1016/0041-0101(89)90146-3

[99] Budzynski AZ, Pandya BV, Rubin RN, Brizuela BS, Soszka T, et al (1984) Fibrinogenolytic afibrinogenemia after envenomation by western diamondback rattlesnake (Crotalus atrox). Blood 63: 1–14. 6537796

[100] Kitchens C, Eskin T (2008) Fatality in a case of envenomation by Crotalus adamanteus initially successfully treated with polyvalent ovine antivenom followed by recurrence of defibrinogenation syndrome. J Med Toxicol 4: 180–183. 18821492 10.1007/BF03161198 PMC3550043

[101] Hardy DL, Jeter M, Corrigan JJ Jr (1982) Envenomation by the northern blacktail rattlesnake (Crotalus molossus molossus): report of two cases and the in vitro effects of the venom on fibrinolysis and platelet aggregation. Toxicon 20: 487–493. 7080054 10.1016/0041-0101(82)90012-5

[102] Hasiba U, Rosenbach LM, Rockwell D, Lewis JH (1975) DiC-like syndrome after envenomation by the snake, Crotalus horridus horridus. N Engl J Med 292: 505–507. 1167934 10.1056/NEJM197503062921004

[103] Bush SP, Green SM, Moynihan JA, Hayes WK, Cardwell MD (2002) Crotalidae polyvalent immune Fab (ovine) antivenom is efficacious for envenomations by Southern Pacific rattlesnakes (Crotalus helleri). Ann Emerg Med 40: 619–624. 12447339 10.1067/mem.2002.129939

[104] Boels D, Hamel JF, Bretaudeau Deguigne M, Harry P (2012) European viper envenomings: Assessment of Viperfav and other symptomatic treatments. Clin Toxicol (Phila) 50: 189–196. 22372786 10.3109/15563650.2012.660695

[105] Petite J (2005) Viper bites: treat or ignore? Review of a series of 99 patients bitten by Vipera aspis in an alpine Swiss area. Swiss Med Wkly 135: 618–625. 16380847 10.4414/smw.2005.11198

[106] Luksic B, Culic V, Stricevic L, Brizic I, Poljak NK, et al (2010) Infant death after nose-horned viper (Vipera ammodytes ammodytes) bite in Croatia: A case report. Toxicon 56: 1506–1509. 20813122 10.1016/j.toxicon.2010.08.009

[107] Mebs D, Holada K, Kornalik F, Simak J, Vankova H, et al (1998) Severe coagulopathy after a bite of a green bush viper (Atheris squamiger): case report and biochemical analysis of the venom. Toxicon 36: 1333–1340. 9723832 10.1016/s0041-0101(98)00008-7

[108] Top LJ, Tulleken JE, Ligtenberg JJ, Meertens JH, van der Werf TS, et al (2006) Serious envenomation after a snakebite by a Western bush viper (Atheris chlorechis) in the Netherlands: a case report. Neth J Med 64: 153–156. 16702615

[109] Hatten BW, Bueso A, French LK, Hendrickson RG, Horowitz BZ (2013) Envenomation by the Great Lakes Bush Viper (Atheris nitschei). Clin Toxicol (Phila) 51: 114–116. 23327286 10.3109/15563650.2012.763134

[110] Lifshitz M, Kastel H, Harman-Boehm I (2002) Cerastes cerastes envenomation in an 18 year old female: a case report. Toxicon 40: 1227–1229. 12165327 10.1016/s0041-0101(02)00124-1

[111] Schneemann M, Cathomas R, Laidlaw ST, El Nahas AM, Theakston RD, et al (2004) Life-threatening envenoming by the Saharan horned viper (Cerastes cerastes) causing micro-angiopathic haemolysis, coagulopathy and acute renal failure: clinical cases and review. QJM 97: 717–727. 15496528 10.1093/qjmed/hch118

[112] Lifshitz M, Kapelushnik J, Ben-Harosh M, Sofer S (2000) Disseminated intravascular coagulation after cerastes vipera envenomation in a 3-year-old child: a case report. Toxicon 38: 1593–1598. 10775758 10.1016/s0041-0101(99)00239-1

[113] Valenta J, Stach Z, Fricova D, Zak J, Balik M (2008) Envenoming by the viperid snake Proatheris superciliaris: a case report. Toxicon 52: 392–394. 18619480 10.1016/j.toxicon.2008.05.021

[114] Jennings BR, Spearman CW, Kirsch RE, Shephard EG (1999) A novel high molecular weight fibrinogenase from the venom of Bitis arietans. Biochim Biophys Acta 1427: 82–91. 10082989 10.1016/s0304-4165(99)00010-0

[115] Warrell DA, Ormerod LD, Davidson NM (1975) Bites by puff-adder (Bitis arietans) in Nigeria, and value of antivenom. Br Med J 4: 697–700. 1203728 10.1136/bmj.4.5998.697 PMC1675831

[116] Lavonas EJ, Tomaszewski CA, Ford MD, Rouse AM, Kerns WP 2nd (2002) Severe puff adder (Bitis arietans) envenomation with coagulopathy. J Toxicol Clin Toxicol 40: 911–918. 12507061 10.1081/clt-120016963

[117] McNally T, Conway GS, Jackson L, Theakston RD, Marsh NA, et al (1993) Accidental envenoming by a Gaboon viper (Bitis gabonica): the haemostatic disturbances observed and investigation of in vitro haemostatic properties of whole venom. Trans R Soc Trop Med Hyg 87: 66–70. 8465400 10.1016/0035-9203(93)90427-r

[118] Porath A, Gilon D, Schulchynska-Castel H, Shalev O, Keynan A, et al (1992) Risk indicators after envenomation in humans by Echis coloratus (mid-east saw scaled viper). Toxicon 30: 25–32. 1595076 10.1016/0041-0101(92)90498-t

[119] Gillissen A, Theakston RD, Barth J, May B, Krieg M, et al (1994) Neurotoxicity, haemostatic disturbances and haemolytic anaemia after a bite by a Tunisian saw-scaled or carpet viper (Echis ‘pyramidum’-complex): failure of antivenom treatment. Toxicon 32: 937–944. 7985198 10.1016/0041-0101(94)90372-7

